# 

**Published:** 1891-12

**Authors:** 


CONTENTS:

PAGE

ORIGINAL ARTICLES:

Echinorhynchus Gigas and its Intermediate Host. By C. W. Stiles, Ph. D. ..... 657
Age of the Hog. By R. S. Huidekoper, M. D., Vet ..... 661
Age of the Dog. By same ..... 670
Preparation of an Animal Suture. By D. A. Johnson, D. V. M. ..... 678
Pilocarpine as a Purgative for the Horse. By same ..... 678
Tattoo for Sheep Marking. By J. B. Hellier ..... 679
Electrical Accidents to Domestic Animals. By James A. Waugh, V.S. ..... 681
Successful Laryngotomy for Laryngismus Paralyticus. By S. J. J. Harger, V. M. D ..... 683
Cases: By J. F. Winchester, D. V. S ..... 684
Torsion of the left Layers of the large Colon in the Horse. By S. J. J. Harger. V. M. D. ..... 687
Review of Recent Publications in Medical Zoology. By C. W. Stiles, Ph. D. ..... 691
The Royal Veterinary College ..... 699

EDITORIAL DEPARTMENT:

United States Veterinary Medical Association ..... 696
Centenaries of Veterinary Schools ..... 698

SOCIETY PROCEEDINGS:

Keystone Veterinary Medical Association ..... 704
Massachusetts Veterinary Association ..... 707
Western Iowa Veterinary Medical Association ..... 709
Western Pennsylvania Veterinary Medical Association ..... 710

REVIEW DEPARTMENT.

Hand Book of Materia Medica, Pharmacy and Therapeutics ..... 710

